# Active neutron and gamma-ray imaging of highly enriched uranium for treaty verification

**DOI:** 10.1038/s41598-017-08253-x

**Published:** 2017-08-11

**Authors:** Michael C. Hamel, J. Kyle Polack, Marc L. Ruch, Matthew J. Marcath, Shaun D. Clarke, Sara A. Pozzi

**Affiliations:** Department of Nuclear Engineering and Radiological Sciences, University of Michigan, 2355 Bonisteel Blvd., Ann Arbor, MI 48109 USA

## Abstract

The detection and characterization of highly enriched uranium (HEU) presents a large challenge in the non-proliferation field. HEU has a low neutron emission rate and most gamma rays are low energy and easily shielded. To address this challenge, an instrument known as the dual-particle imager (DPI) was used with a portable deuterium-tritium (DT) neutron generator to detect neutrons and gamma rays from induced fission in HEU. We evaluated system response using a 13.7-kg HEU sphere in several configurations with no moderation, high-density polyethylene (HDPE) moderation, and tungsten moderation. A hollow tungsten sphere was interrogated to evaluate the response to a possible hoax item. First, localization capabilities were demonstrated by reconstructing neutron and gamma-ray images. Once localized, additional properties such as fast neutron energy spectra and time-dependent neutron count rates were attributed to the items. For the interrogated configurations containing HEU, the reconstructed neutron spectra resembled Watt spectra, which gave confidence that the interrogated items were undergoing induced fission. The time-dependent neutron count rate was also compared for each configuration and shown to be dependent on the neutron multiplication of the item. This result showed that the DPI is a viable tool for localizing and confirming fissile mass and multiplication.

## Introduction

Treaty verification of special nuclear material (SNM) (plutonium, or uranium enriched in ^235^U or ^233^U) demands strategies that can identify key characteristics of an item, such as the energy of emitted neutron and gamma-ray radiation, and its enrichment or multiplication^1–3^. The chosen means of verification must also present a robust method that eliminates opportunities for *spoofing*, which is the attempt to pass off a different material or radioisotope as the expected SNM item. Materials with a response similar to SNM could be used as a *hoax* ^4^.

Verification of highly enriched uranium (HEU) presents a unique challenge due to its low neutron emission rate and emission of characteristic gamma rays that are low energy and easily shielded. HEU is uranium that has been enriched to greater that 20% ^235^U, with weapons-grade uranium enriched to at least 90%^5, 6^. Using active interrogation to induce fission in the HEU can help overcome these limitations by producing a significant neutron emission rate and creating more high energy gamma rays from the induced fission events. Interrogation with a deuterium-tritium (DT) neutron generator is a method used to induce fission in HEU^7^. The generator produces 14.1-MeV neutrons through the fusion of deuterium and tritium.

While active interrogation is well established for producing a measurable signal of neutrons and gamma rays, much of the available literature simply examines the increased count rate compared to the background or active background^7–9^. In these cases, positive detection can be susceptible to large fluctuations in the environmental or active background. The latter is the signal produced by the generator when no item is present for interrogation. Using only count rates provides opportunities for spoofing because the detected particles contributing to an increased count rate cannot be directly attributed to the interrogated item; Mating active interrogation with radiation imaging addresses this issue by spatial localization of the item. This approach provides an increased signal-to-background ratio.

Some efforts to combine imaging and active interrogation have been made with coded aperture and scatter cameras. Coded aperture cameras were used to image thermal neutrons in depleted uranium (DU)^10^ and used to detect HEU interrogated by a DT neutron generator, but achieved limited success^11^. The neutron scatter camera from Sandia National Laboratories (SNL) measured low-enriched uranium (LEU), and the MINER system, also from SNL, was used to create an image of HEU actively interrogated with AmLi^12, 13^; however, none of these efforts have sought to include neutron spectroscopy or analysis of source multiplication to improve verification.

Other related active interrogation methods are the Nuclear Material Identification System and the Advanced Portable Neutron Imaging System, both developed by Oak Ridge National Laboratory^14, 15^. These systems use a related technique known as associated particle imaging, in which the direction of each interrogating neutron can be tracked. While both systems are able to verify characteristics of HEU, they require the added complexity of the associated particle method and for the sample to be located between the generator and detector array. These systems also do not include a mechanism to perform fast neutron spectroscopy.

**Figure 1:**
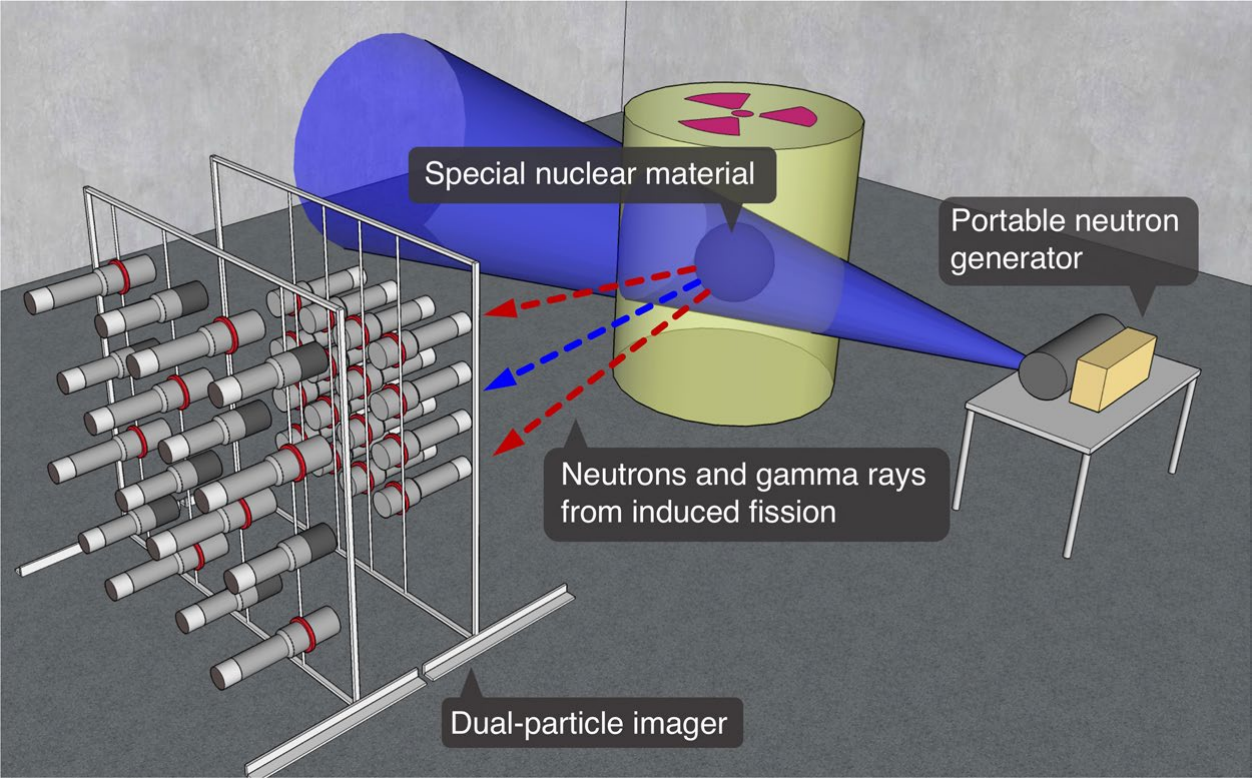
A schematic of a possible warhead verification scenario using the DPI and a portable DT neutron generator.

This work presents a new method designed to verify characteristics of HEU and to provide more certainty in a verification setting. We propose using the dual-particle imager (DPI), which is capable of localizing both neutrons and gamma rays separately, as well as performing spectroscopy on detected particles^16, 17^. The imager is paired with a commercial, portable neutron-generator to induce fission in the HEU with 14.1-MeV neutrons; Fig. 1 shows a schematic of a hypothetical verification scenario using these instruments. This work also demonstrates how the time-dependent neutron count rate can be used to infer the relative multiplication of samples, which is related to the fissile mass and enrichment^18, 19^. The time-dependent neutron count rate is defined as the distribution of neutron detection times occurring in the system relative to each generator pulse.

To demonstrate the viability of this method, experiments using a 13.7-kg sphere of HEU enriched to 93.16% ^235^U were performed at the Device Assembly Facility, which is located at the Nevada National Security Site. Three configurations with the HEU were measured, including: no moderation, 3.81 cm of low-Z high-density polyethylene (HPDE) moderation, and 2.54 cm of high-Z tungsten moderation. A tungsten object, representing a possible hoax item, was also interrogated. The results obtained from these experiments include reconstructed neutron and gamma-ray images that localize the interrogated item, estimates of the emitted neutron spectra, and analyses of the time-dependent neutron count rate. Finally, simulated results are used to demonstrate differences in the time-dependent neutron count rate between an HEU and a DU sample.

## Results

### Localization of weapons-grade HEU

In all measured configurations, a DT neutron generator, with an approximate output of 7 × 10^7^ neutrons per second, was placed on a table adjacent to the target HEU configuration at the same height. The DT neutron generator produced a logic pulse to designate the start time of each neutron pulse. Each configuration was first evaluated using the correlated count rate recorded by the DPI and the reconstructed neutron and gamma-ray images. In this context, the correlated count rate refers to neutron and gamma-ray events that have double-scattered in the system to create an imagable event. Methods for reconstructing separate neutron and gamma-ray images using the DPI have been previously documented^16, 17^.

Table 1 shows a comparison of the correlated count rates. To ensure that the HEU fission neutrons and gamma rays were contributing to the correlated count rate, and not direct neutrons from the DT neutron generator, a veto was applied such that any counts recorded during the pulse were discarded. The table shows that the correlated neutron count rate from the bare HEU was several orders of magnitude higher than when no item was present. In this same example, the correlated gamma-ray count rate increased 21% relative to the case with no item present.

**Table 1:** The correlated count rates for each experimental configuration.

Item	Moderation	Neutrons per s.	Gamma rays per s.
None	N/A	0.0048 ± 0.0034	85.8 ± 0.5
13.7 kg HEU	None	0.217 ± 0.008	103.5 ± 0.2
13.7 kg HEU	HDPE	1.34 ± 0.03	130.5 ± 0.3
13.7 kg HEU	Tungsten	0.195 ± 0.018	95.8 ± 0.4
Tungsten sphere	None	0.018 ± 0.006	85.8 ± 0.4

**Figure 2:**
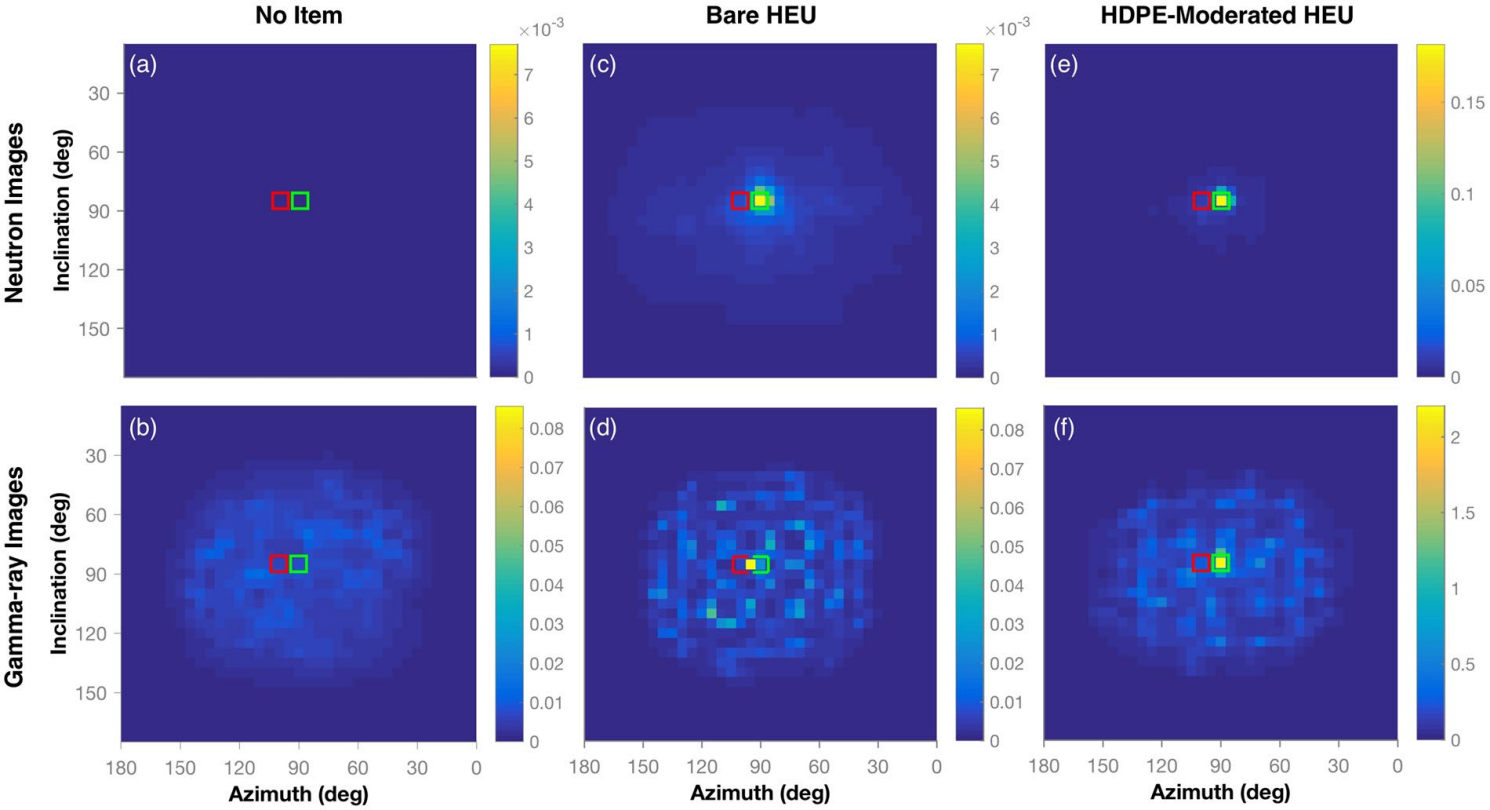
A comparison of the reconstructed neutron and gamma-ray images from the configuration with no item, bare HEU, and HDPE-moderated HEU. The scales for all images are in units of correlated counts per second. The overlaid red and green boxes represent the locations of the DT neutron generator and HEU, respectively. When no item is present, the neutron image (**a**) and gamma-ray image (**b**) do not show any source. When the HEU is interrogated, it can be located with neutrons (**c**) and gamma rays (**d**). When an HDPE moderator is added, neutron and gamma-ray emissions are increased relative to the bare case, providing greater signal-to-noise ratio in the resulting neutron (**e**) and gamma-ray (**f**) images.

The reconstructed images for the case with no item, the bare HEU, and HDPE-moderated HEU are compared in Fig. 2. For this work, the stochastic origin ensembles image reconstruction technique was chosen due to the high quality of reconstructed images^20^. The neutron and gamma-ray images for the bare HEU case, Fig. 2(c) and (d) respectively, were scaled for maximum contrast between the maximum and minimum pixel value. For the case with no item, the images were displayed using the same scale as when the bare HEU sphere was used as the target. This scale helps to highlight the increased correlated count rates from the HEU target. In all images, a red and green box are overlaid to show the positions of the DT neutron generator and target, respectively. The scale values in all cases are in units of correlated counts per second.

The neutron image reconstructed of the bare HEU shows a hot-spot located within the green box and very small contribution from the red box. The location of the hot-spot provides confidence that the neutrons imaged were from the HEU and not the DT neutron generator. The result shows that the technique is able to correctly image the HEU. To create a well defined hot-spot from the gamma rays, a lower energy threshold of 2.5 MeV was applied with an upper threshold of 2.8 MeV. This threshold removes a large contribution from the environmental background, and lower energy fission gamma rays, but not the high energy fission gamma rays. The upper threshold removes events that reconstruct poorly due to system resolution effects. The hot-spot is not centered in the green box; it appears in the pixel between the red and green box. To directly compare the gamma-ray image with no item present (Fig. 2(b)) to the image of the bare HEU configuration (Fig. 2(d)), the 2.5-MeV lower and 2.8-MeV upper thresholds were also applied. The comparison shows that the inclusion of bare HEU produces the hot-spot in Fig. 2(d), despite it not occurring in the expected pixel. We believe that the small error in localization is statistical in nature and not due to a systematic bias. A low signal-to-noise ratio and limited number of correlated gamma rays detected from the HEU likely produced the error. The correlated count rate with the applied energy window with the HEU present was 3.2706 ± 0.0304 and was 3.0905 ± 0.0858 with no item. These correlated count rates provide an estimate that only 638 ± 322 total counts were measured from the HEU in the 59 minute measurement while 10940 ± 304 total counts were from the active background.

#### Moderated HEU

Two different moderated configurations, one with low-Z HDPE and another with high-Z tungsten, were used to evaluate how the presence of shielding could affect detection of the HEU. Comparing the correlated count rates to the bare case in Table 1, the presence of the HDPE around the HEU increases both the neutron and gamma-ray rates. This increase is especially significant for gamma-ray localization. In Fig. 2(f), the gamma-ray hot-spot is located in the correct pixel and there is visibly less noise compared to the bare HEU image. In this case, the correlated count rate was significant enough compared to background to apply a lower threshold of only 0.5 MeV. The 0.5-MeV threshold is applied in this case because low energy events do not reconstruct well due to the configuration of the DPI. The neutron image from the HDPE-moderated case also reflects the increased correlated count rate. The HEU is correctly localized and there is less noise in the image than for the bare HEU image. The image scales for both HDPE-moderated HEU images were set to achieve maximum contrast and differ from the scales used for the case with no item and bare HEU. A comparison can be made by comparing pixel values, given in correlated counts per second.

The increased correlated count rates seen in the HDPE-moderated case is due to the configuration having a larger multiplication. The HDPE shell moderates and reflects neutrons emitted from the HEU back into the sphere where they induce more fission reactions. To investigate the difference in the effective multiplication factor, *k*_*eff*_ is calculated. The quantity *k*_*eff*_ is a measure of the neutron population in a generation divided by the neutron population in the previous generation. The MCNP6 KCODE was used to calculate *k*_*eff*_ for the bare and HDPE-moderated configurations^21^. The bare 13.7-kg HEU sphere had a *k*_*eff*_ of 0.6495 ± 0.0003 while the HDPE-moderated HEU configuration had a *k*_*eff*_ of 0.7642 ± 0.0006.

The tungsten-moderated HEU configuration did not produced an increased correlated count rate (Table 1). Instead, the tungsten shell acted as an attenuator for neutrons entering and leaving the sphere, which decreased the correlated neutron count rate by 10% relative to the bare case. The high-Z tungsten also depressed the correlated gamma-ray count rate such that it was only slightly increased from the measurement with no item present. The measurement time for this configuration was not long enough to collect an adequate number of correlated counts for good image reconstruction; however, it is reasonable to expect that with adequate statistics, the neutron image would appear similar to the neutron image from the bare HEU measurement. The slightly elevated correlated gamma-ray count rate was not sufficient to locate the HEU.

#### Hoax item

A hollow tungsten sphere, similar in size to the HEU, was used to represent a hoax item. The correlated neutron and gamma-ray count rates were not large enough to localize the tungsten sphere and were similar to the correlated count rates when no item was present. This result was important to show that a heavy-metal object would not produce a false positive in the system; however, a DU hoax would undergo induced fission and produce a localized signal. The next two sections address other DPI measurables that provide improved certainty in verification of the interrogated material.

### Fast neutron spectroscopy

**Figure 3:**
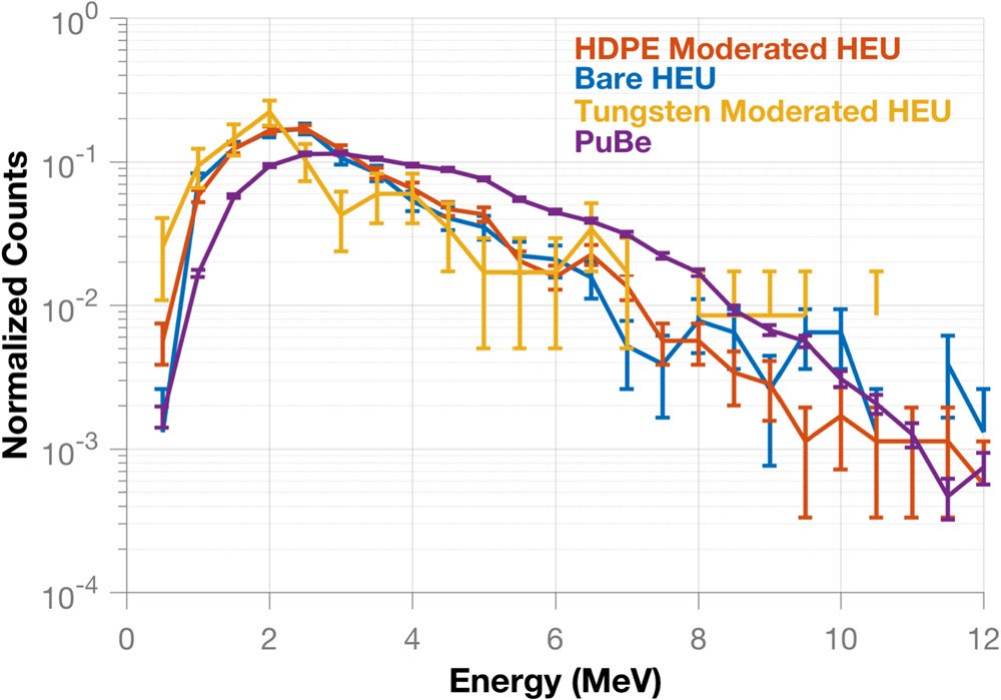
Normalized neutron spectra for each configuration are compared to a measured PuBe spectrum. This comparison highlights the difference in the reconstructed spectra created from pure induced fission sources and the PuBe, which emits more high energy neutrons though (*α*, n) reactions. The reconstructed spectra reflect the difference between the Watt and (*α*, n) spectra. The configurations with HEU all follow the same trend and have a larger contribution from lower energy neutrons than the PuBe spectrum.

Neutron spectra for the bare and moderated HEU configurations given in Table 1 are compared in Fig. 3. The spectra are created from the correlated counts recorded in the DPI. A spectrum from the measurement of a PuBe source, in a similar position to the HEU, is included to highlight the difference in reconstructed spectra measured from different emitted neutron spectra. All spectra have been integral normalized to allow for a comparison of the shape. The neutron spectrum from induced fission in the HEU has the shape of a Watt spectrum while the neutrons emitted by PuBe, through the (*α*, n) reaction, create a spectrum with more contribution at higher energies in comparison. This comparison demonstrates the ability of the DPI to detect spectral differences for fast neutrons.

While localizing and verifying the neutron spectral shapes provides knowledge that the item is producing neutrons from fission, a hoax item made from DU or LEU would also produce fission neutrons and gamma rays from induced fission events in the ^238^U. In this case, the correlated count rate would be lower because the cross-section for induced fission is lower for ^238^U than ^235^U. To address this issue, the next section explores using the time-dependent neutron count rate to differentiate among items having varying multiplication.

### Time-dependent neutron count rate analysis

**Figure 4:**
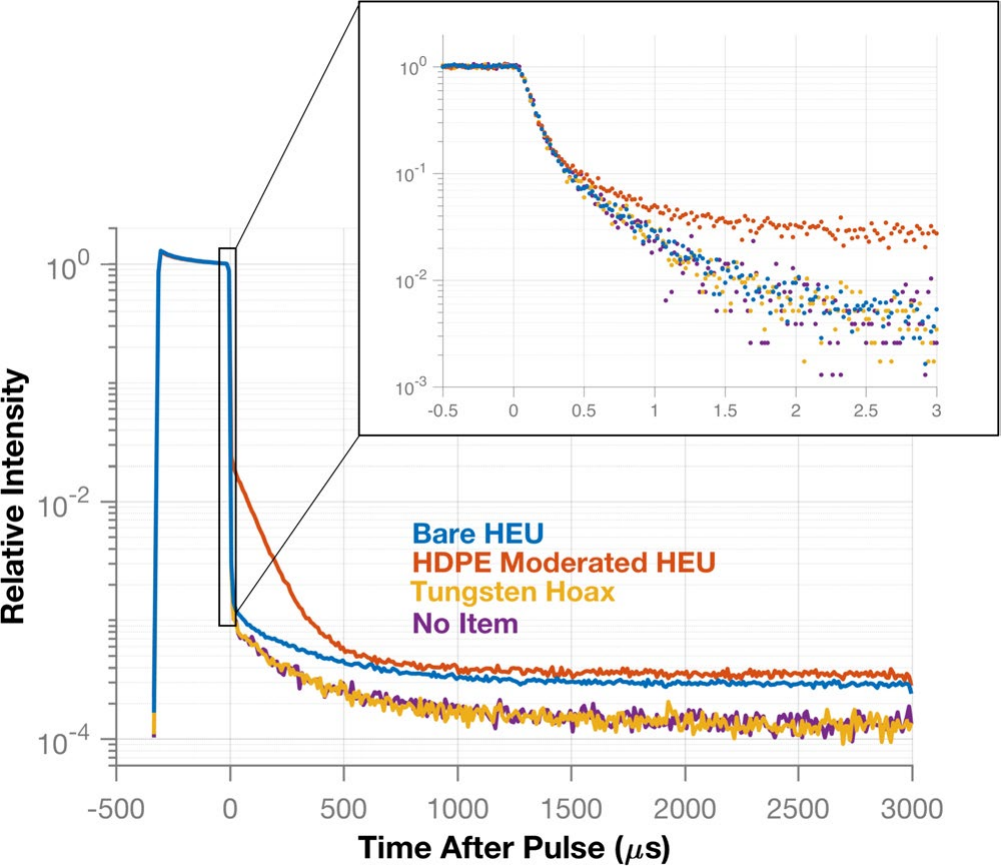
The time-dependent neutron count rate for the measured configurations is compared. The main plot shows the entire structure of a pulse and the time between pulses while the inset plot shows the decay behavior in the first 3 μs using a finer binning. The HDPE-moderated HEU shows a slower initial decay while the other cases are indistinguishable. However, when the full time scale of a pulse is examined, there is a difference between the bare HEU and the no item/tungsten hoax cases. The count rate distribution for the tungsten hoax follows that of the active background showing that a non-multiplying source can be discriminated from a multiplying one.

Once localization has verified that neutron counts are from the interrogated item, the time-dependent neutron count rate can be analyzed to characterize the multiplication of the item. The rate at which the time-dependent neutron count rate decays after a generator pulse corresponds to the fissile multiplication of an item. The distribution of the time-dependent neutron count rate is created by histogramming the detection times for neutron counts acquired by the 16 front plane organic liquid scintillators. This detector type was used previously for neutron multiplication analysis, which demonstrated that the excellent 1-ns timing resolution was sufficent to record the behavior of time-dependent fast neutron data^19^. For clarity it is important to mention that the time-dependent neutron count rate uses neutrons detected in a single detector and is different than the previously discussed correlated count rate. Figure 4 shows a comparison of the time-dependent neutron count rate for four different configurations. The main plot shows the decay of the count rate over the entire period after a pulse, with a bin width of 10 μs, while the inset plot shows the decay within the first 3 μs after the pulse, with a bin width of 20 ns.

During the first 3 μs, the neutron count rate from the HDPE-moderated HEU clearly decays at a slower rate than the bare HEU, tungsten hoax, and the active background. Neutrons emitted by the HEU are reflected by the HDPE back into the HEU, which causes an increased number of induced fissions and therefore a slower decay of the neutron count rate compared to the bare configuration. The initial component of the count rate decay does not appear sufficient to distinguish between the bare HEU, the hoax, or when there is no item. However, a slower decay is seen in the full count rate distribution of the bare HEU configuration compared to that of the active background. For the tungsten hoax, the count rate decay is the same as the active background, showing that no multiplication is occurring in the item.

#### Simulated results showing discrimination of HEU and DU

An identical item consisting of DU or LEU could possibly be used to spoof the system. DU and LEU by definition have a lower ^235^U enrichment than HEU and will have a lower multiplication. Under DT neutron interrogation, DU would undergo induced fission, which would allow for localization and produce a neutron Watt spectrum. However, because the multiplication is much smaller in DU compared to HEU, the time-dependent neutron count rate will decay faster in DU compared to HEU. Because a DU sample was not available for the experiments, a Monte Carlo simulation was performed using the experimental setup to evaluate the count rate decay.

**Figure 5:**
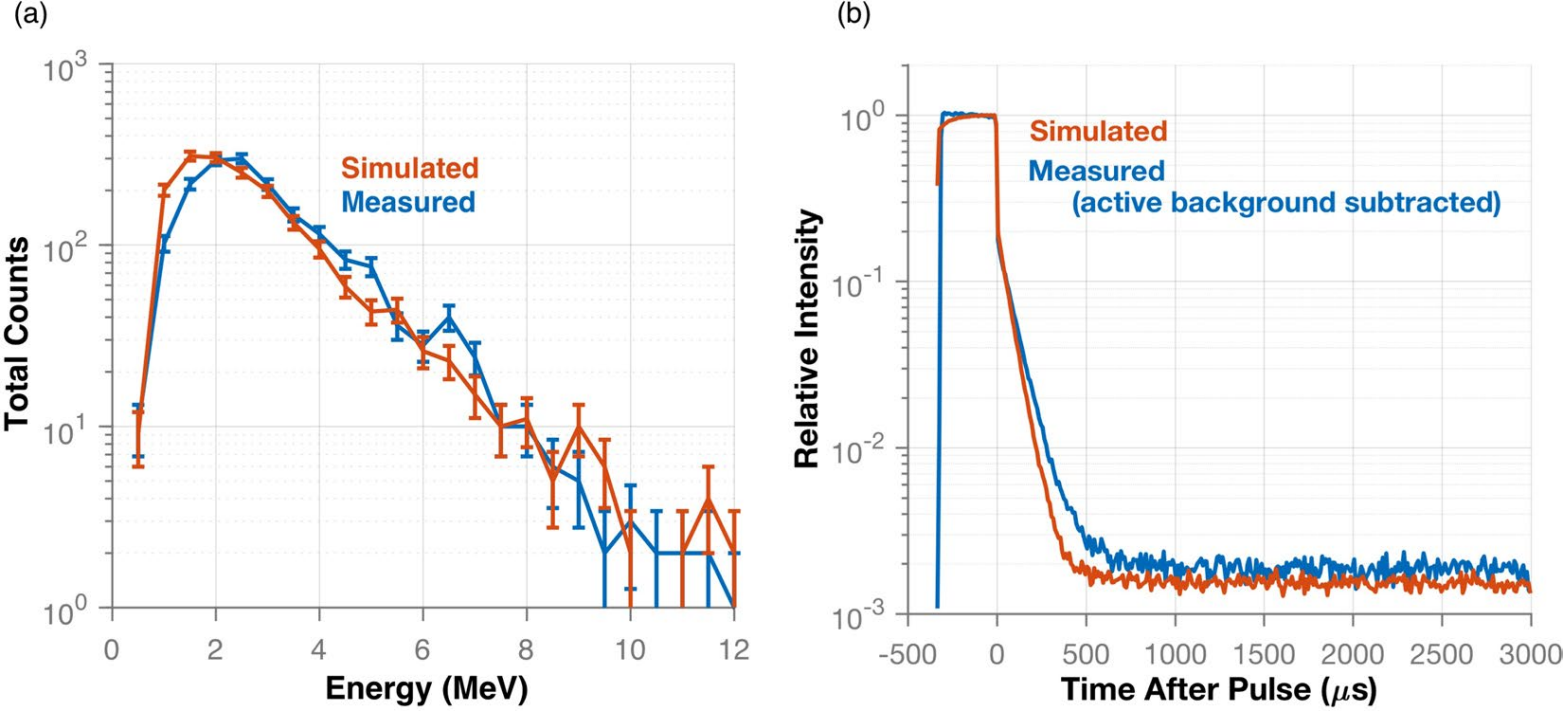
Simulated and measured results are compared for the experiment using 3.81 cm of HDPE to moderate the HEU. The fast neutron spectra (**a**) shown good agreement with the total count rate differing by 1.9%. The time-dependent neutron count rates (**b**) appear similar as well.

To validate the Monte Carlo simulation results, a comparison was made between measurement and simulation. In Fig. 5(a) the reconstructed fast neutron spectra were compared for the HEU configuration moderated with 3.81 cm of HDPE. The magnitude and shape of the spectra agree well with the simulation, producing a correlated count rate that was 1.9 ± 3.3% greater than the measurement. Most of the disagreement in the spectra is seen at low energies and may be attributable to model mismatch in the photomultiplier tubes (PMTs). The modeled PMTs may not attenuate a sufficient amount of low energy neutrons, which would have less of an effect on higher energy neutrons.

Figure 5(b) compares the time-dependent neutron count rate for the HDPE-moderated configuration. To make a direct comparison, the active background count rate distribution was subtracted from the count rate distribution measured with the HPDE-moderated HEU. This background subtraction was necessary because the simulation emitted particles from the neutron generator in a cone directed towards the item instead of isotropically, which greatly reduced computation time. The simulated time-dependent neutron count rate decays faster than the measured distribution. One possible explanation for this occurrence is a difference in the composition of the HDPE used in the measurement and the simulation. If the HDPE were more dense in the measurement than the simulation, more neutrons would be reflected back into the HEU creating induced fission events later in time after the pulse.

Simulations of the other configurations were performed to compare correlated neutron count rates with measured results. For the bare HEU configuration, the simulated correlated count rate was 10 ± 5% greater than the measured result. The tungsten-moderated simulation produced a correlated count rate 27 ± 13% greater than the measurement. The large error in this correlated count rate difference was due to a short measurement time. Because the HDPE-moderated case provided the best agreement between measurement and simulation, the same moderation was used to compare the time-dependent neutron count rate for the simulation of HEU and a simulation using a DU sphere of the same size.

**Figure 6:**
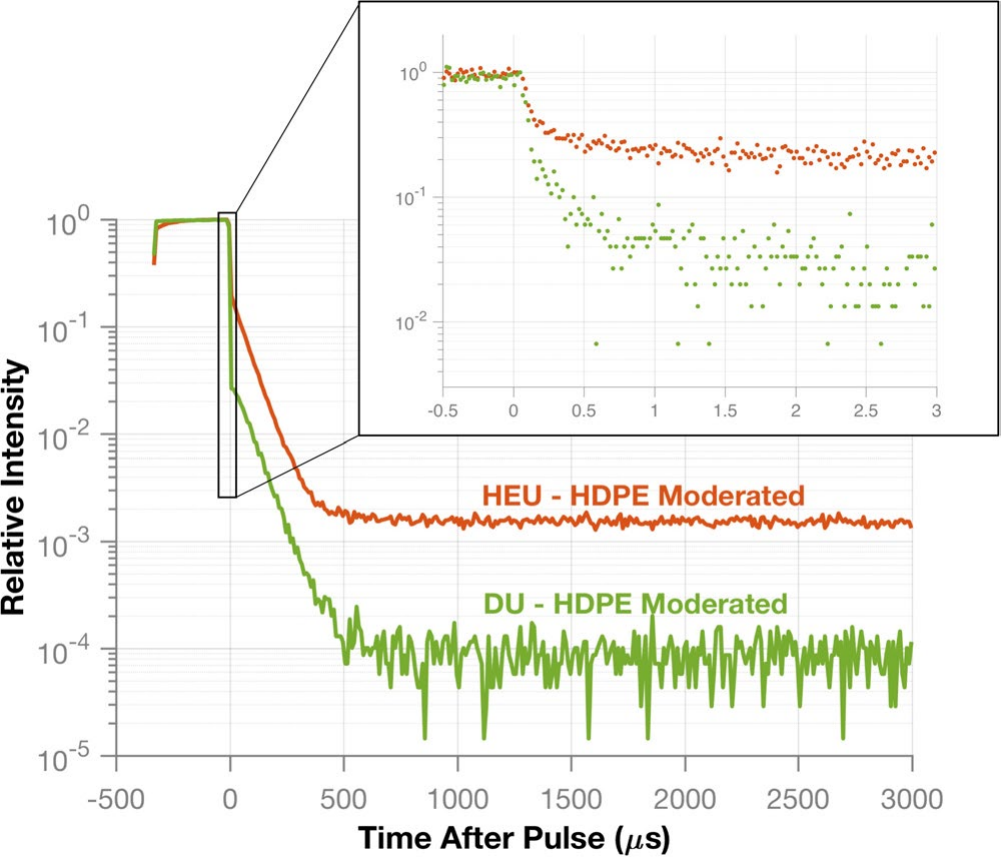
The simulated time-dependent neutron count rate is compared for HEU and DU both moderated by HDPE. Both the entire pulse structure and the first 3 μs (inset) show a difference between the count rate decay for the HEU and DU. The HEU count rate decays at a slower rate than the DU due to a higher probability of induced fission.

An MCNP6 KCODE calculation showed that the *k*_*eff*_ of the HDPE-moderated DU sphere was 0.1447 ± 0.0001. This value is much lower than the value for the HDPE-moderated HEU sphere (0.7642 ± 0.0006). A comparison of the time-dependent neutron count rate is shown in Fig. 6. In the simulation, a cone of 14.1-MeV neutrons was focused on the target to improve simulation time compared to an isotropic source. As a result, neutrons detected by the DPI due to direct shine from the DT neutron generator are not included in the histogram. It is clear by examining both the first 3 μs and the entire histogram that the time-dependent neutron count rate for HEU and DU, both moderated by HDPE, are distinctly different. In both cases, the HEU displays a slower count rate decay than the DU.

## Discussion

This work demonstrates that the DPI paired with a DT neutron generator can determine the location and identify key properties of an interrogated item needed to verify that an item is HEU. We showed that a source-to-system distance of 155 cm and a generator output of approximately 7 × 10^7^ neutrons per second was sufficient for locating 13.7 kg of HEU in one hour or less depending on the moderation. A 3.81-cm HDPE moderator made the correlated neutron count rate 6.18 times larger and the background-subtracted correlated gamma-ray count rate 2.53 times larger than the respective correlated count rates for the bare configuration. A 2.54-cm tungsten moderator only decreased the correlated neutron count rate by 10% and decreased the background-subtracted correlated gamma-ray count rate by 44%. When a large tungsten object was interrogated, the correlated neutron and gamma-ray count rates were not significantly increased above the active background, providing confidence that neutrons from the generator scattering off of a heavy metal object would not produce a significant signal in the system. Monte Carlo simulations of the experiments were validated by comparison to measured correlated count rates. The agreement achieved was 10%, 1.9%, and 27% respectively for bare, HDPE-moderated, and tungsten-moderated cases.

With source localization achieved, the reconstructed neutron spectra could be attributed to the items and were evaluated. The spectra from the HEU configurations were compared with the (*α*, n) spectrum measured from a PuBe source. This comparison showed the ability of the system to discriminate between different types of neutron spectra. Finally, the time-dependent neutron count rate was evaluated to infer differences in multiplication. The first 3 μs after the generator pulse showed a distinct difference between the HDPE-moderated HEU and the other configurations. A longer time scale was required to show the difference in count rate decay for the bare HEU configuration compared to the configurations with no HEU. Simulated results were used to demonstrate the difference in count rate decay for an HEU and DU sphere of the same size. The slower count rate decay for the HEU case was evident in the first 3 μs after the generator pulse. These results presented a framework for verifying a large quantity of HEU and succeeded in eliminating several possible spoofing scenarios. This approach has application in nuclear treaty verification.

Several extensions to this work can be made to improve verification capabilities. The resolution of the DPI could be improved to allow for the shape of the item to be evaluated instead of appearing as a point source. Moving the system closer to the interrogated item would increase the signal-to-background ratio for gamma rays, which could provide for their spectral analysis. To detect more induced fission particles, the use of a collimated DT source would allow for continuous interrogation (without pulsing) and all measured counts could be attributed to the HEU without use of a veto. Finally, a more rigorous characterization of the time-dependent neutron count rate must be performed and tested with items of differing mass and enrichment, which would allow for conclusions to be drawn about the mass of ^235^U in an item.

## Methods

### Description of HEU sample and moderators

Experiments with HEU were carried out at the Device Assembly Facility, which is located within the Nevada National Security Site. The HEU samples used are known as the *Rocky Flats Shells*, which consist of multiple hemispheres that can be configured to achieve different masses of HEU^22^. The sample was enriched to 93.16% ^235^U with a mass of 13.7-kg. The full isotopic composition was: ^234^U, 1.02 wt%; ^235^U, 93.16 wt%; ^236^U, 0.47 wt%; ^238^U, 5.35 wt%^22^. The outer radius of the sphere measured 5.7 cm.

### Experimental configurations

**Figure 7:**
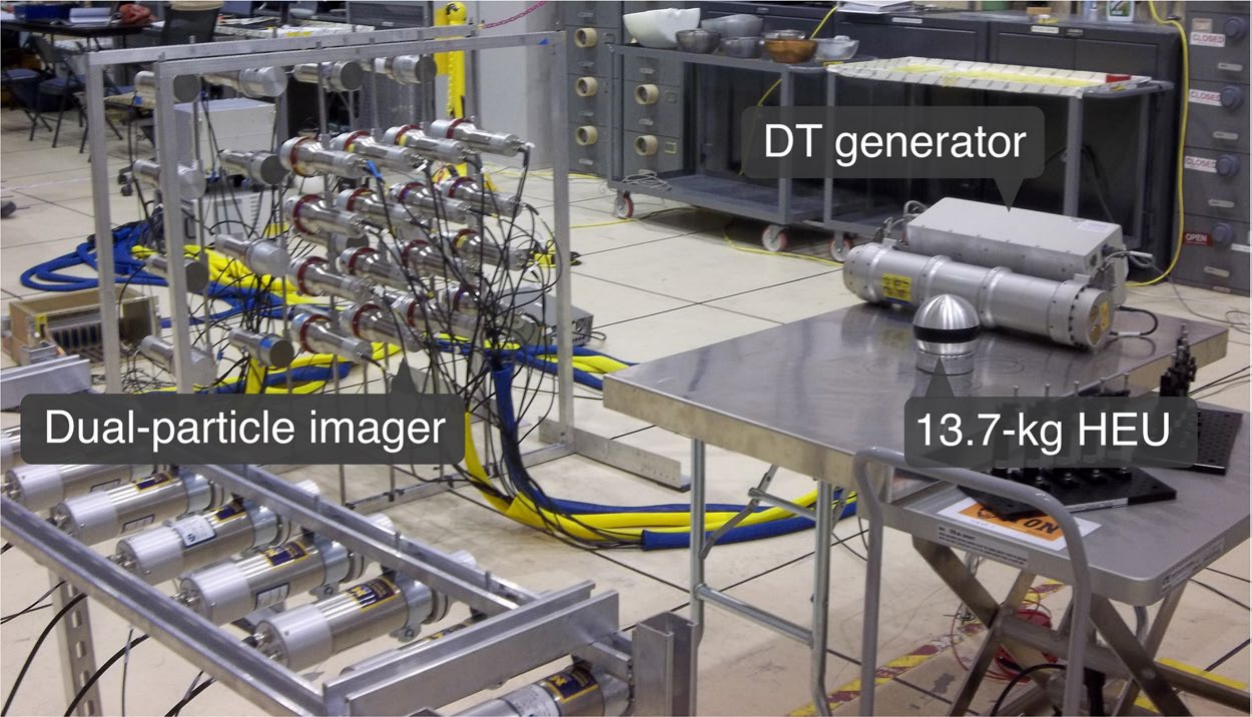
A photograph of the experimental setup that shows the configuration used to measure the HEU interrogated by the DT neutron generator.

The DT neutron generator was placed on an aluminum table at a distance of 158 cm from the center of the DPI. The target in the DT neutron generator had angular coordinates of (100°, 86°). The sample being interrogated was placed on the table with the sample center at a distance of 155 cm and angular coordinates of (90°, 86°). The bare configuration was measured for 59 minutes. The case with no item present was measured for seven minutes. Figure 7 shows a photograph of the experimental setup for the bare HEU configuration.

The HDPE moderator was a 3.81-cm thick shell, which left a 2-cm gap between the inside of the shell and the HEU when surrounding it. A 22 minute measurement was taken with the HDPE moderator. The tungsten moderator was 2.54-cm thick and left a 0.7-cm gap between the HEU and shell. This configuration was measured for 10 minutes. The HEU was removed from the tungsten moderator to create the hoax target. This configuration was also measured for 10 minutes.

### Operation of the DT neutron generator

The experiments used a Thermo Scientific MP 320 neutron generator to interrogate the HEU. The neutron generator was configured with the DT tube, which produced 14.1-MeV neutrons. The generator was set to a pulse length of 3.33 μs with a repetition rate of 300 Hz. These parameters defined the duty cycle to be 10%. The current was operated at approximately 50 μA with a high voltage of 70 kV.

Work by Remetti *et al*. was used to evaluate the anisotropy present in the neutron emission from the generator, which provides an estimate of the total emission, for different current and voltage settings, as well as the angular emission^23^. Their work suggests that a voltage 70 kV and a current of 50 μA will produce a neutron yield of about 6 × 10^7^ neutrons per second. By the coordinate definitions in the paper, the HEU sample was located at 0° for the following experiments, which was stated to have a 15% greater flux than the total emission. Based on these estimates, assuming an isotropic emission of approximately 7 × 10^7^ neutrons per second produces the proper neutron flux in the direction of the HEU.

### The dual-particle imager

The DPI is a two-plane detection system that combines a Compton and neutron scatter camera. The front plane is a 4 × 4 square array of EJ-309 organic liquid scintillator cells. These detectors are 5.1-cm thick with a 7.6-cm diameter and have excellent pulse shape discrimination capability. The back plane is a 4 × 4 square array of EJ-309 and NaI(Tl) scintillators arranged in a checkerboard pattern. Both the EJ-309 and NaI(Tl) scintillators are 7.6-cm thick with a 7.6-cm diameter. The front plane detectors had a detector center-to-center spacing of 15 cm while the back plane had a larger spacing of 25 cm. The planes were separated by 30-cm measured from the back plane detector faces to the front plane detector faces. A full description of the DPI configuration and the physics principles used can be found in work by Poitrasson-Rivière *et al*.^16, 17^. Signals from the DPI were digitized using three CAEN V1730 digitizers. Coincidence logic was implemented to prevent large data acquisition rates that would cause significant digitizer deadtime.

The generator produced a TTL logic pulse that was input to a signal generator. The pulse was converted to a positive, square pulse with an amplitude of 1 V and the signal was connected to one channel in the DPI digitizers resulting in time synchronization with detector pulses. To record the start time of a pulse, the leading edge of the TTL logic pulse was digitized. Data were continuously acquired by the system, even while the generator was on. The digitized generator signal was used to apply a veto in post processing, with a length of 335,700 ns after the beginning of the signal.

The DPI algorithms reconstruct the emitted energy of fast neutrons by summing the energy deposited in a front plane liquid organic scintillator with the energy calculated from the time-of-flight between the two required interactions. Each scatter occurs in a separate organic liquid scintillator, which provides sufficient energy and time resolution to achieve a reasonable estimate for the incident energy of a particle.

To evaluate the time-dependent neutron count rate, the detection times for neutrons detected in the 16 front-plane liquid scintillators were histogrammed based on the start time of the generator pulse. For each spectrum, the value at time zero (the end of the generator pulse) is normalized to a value of one.

### Monte Carlo simulation of the dual-particle imager

The Monte Carlo radiation transport code MCNPX-PoliMi was used for simulation^24^. The source neutrons from the DT neutron generator were distributed in time, matching the pulse structure of the generator. This approach required the accurate simulation of delayed neutrons and the application of a pulse veto for the simulation to be equivalent to the experiments. The source was time distributed with particles only emitted between the start and stop times for each pulse. To limit the number of source particles simulated, the 14.1-MeV neutrons were directed in a cone towards the HEU instead of isotropically. The delayed neutron option was turned on and cross sections from the ENDF/B-VI.2 library were used for all uranium isotopes.

The veto was applied to the MCNPX-PoliMi collision files by removing any interactions with a time stamp that occurred from the start of a pulse to 335,700 ns after the pulse. The collision files were then processed with MPPost to create the DPI response^25^. A more complete description of how the system response of the DPI is simulated is provided by Hamel *et al*.^26^.
