# Author Correction: Active neutron and gamma-ray imaging of highly enriched uranium for treaty verification

**DOI:** 10.1038/s41598-018-32882-5

**Published:** 2018-10-03

**Authors:** Michael C. Hamel, J. Kyle Polack, Marc L. Ruch, Matthew J. Marcath, Shaun D. Clarke, Sara A. Pozzi

**Affiliations:** Department of Nuclear Engineering and Radiological Sciences, University of Michigan, 2355 Bonisteel Blvd., Ann Arbor, MI 48109 USA

Correction to: *Scientific Reports* 10.1038/s41598-017-08253-x, published online 11 August 2017

This Article contains a typographical error in the Methods section under subheading ‘Operation of the DT neutron generator’ where,

“The generator was set to a pulse length of 3.33 μs with a repetition rate of 300 Hz.”

should read:

“The generator was set to a pulse length of 333.33 μs with a repetition rate of 300 Hz.”

Additionally, in the Results section under the subheading ‘Localization of weapons-grade HEU’,

“The tungsten-moderated HEU configuration did not produced an increased correlated count rate (Table 1).”

should read:

“The tungsten-moderated HEU configuration did not produce an increased correlated count rate (Table 1).”

